# Comparative outcomes of obese and non-obese patients with lumbar disc herniation receiving full endoscopic transforaminal discectomy: a systematic review and meta-analysis

**DOI:** 10.1186/s12891-024-07455-5

**Published:** 2024-04-23

**Authors:** An-Ping Feng, Shang-Feng Yu, Chien-Min Chen, Li-Ru He, Shang-Wun Jhang, Guang-Xun Lin

**Affiliations:** 1 Department of Orthopedics and Traumatology of Traditional Chinese Medicine, The Third Hospital of Xiamen, Xiamen, China; 2 Department of clinical laboratory, The Third Hospital of Xiamen, Xiamen, China; 3 Division of Neurosurgery, Department of Surgery, Changhua Christian Hospital, Changhua, Taiwan; 4 Department of Leisure Industry Management, National Chin-Yi University of Technology, Taichung, Taiwan; 5 Department of Biomedical Sciences, National Chung Cheng University, Chiayi, Taiwan; 6 Department of Anesthesia and Surgery, The first affiliated Hospital of Xiamen University, Xiamen University, Xiamen, Fujian China; 7 Department of Orthopedics, School of Medicine, The First Affiliated Hospital of Xiamen University, Xiamen University, Xiamen, China; 8 The Third Clinical Medical College, Fujian Medical University, Fuzhou, Fujian China

**Keywords:** Obese, Full endoscopic spine surgery, Transforaminal, Disc herniation, BMI

## Abstract

**Objective:**

This study aimed to assess the impact of full endoscopic transforaminal discectomy (FETD) on clinical outcomes and complications in both obese and non-obese patients presenting with lumbar disc herniation (LDH).

**Methods:**

A systematic search of relevant literature was conducted across various primary databases until November 18, 2023. Operative time and hospitalization were evaluated. Clinical outcomes included preoperative and postoperative assessments of the Oswestry Disability Index (ODI) and visual analogue scale (VAS) scores, conducted to delineate improvements at 3 months postoperatively and during the final follow-up, respectively. Complications were also documented.

**Results:**

Four retrospective studies meeting inclusion criteria provided a collective cohort of 258 patients. Obese patients undergoing FETD experienced significantly longer operative times compared to non-obese counterparts (*P* = 0.0003). Conversely, no statistically significant differences (*P* > 0.05) were observed in hospitalization duration, improvement of VAS for back and leg pain scores at 3 months postoperatively and final follow-up, improvement of ODI at 3 months postoperatively and final follow-up. Furthermore, the overall rate of postoperative complications was higher in the obese group (*P* = 0.02). The obese group demonstrated a total incidence of complications of 17.17%, notably higher than the lower rate of 9.43% observed in the non-obese group.

**Conclusion:**

The utilization of FETD for managing LDH in individuals with obesity is associated with prolonged operative times and a higher total complication rate compared to their non-obese counterparts. Nevertheless, it remains a safe and effective surgical intervention for treating herniated lumbar discs in the context of obesity.

**Supplementary Information:**

The online version contains supplementary material available at 10.1186/s12891-024-07455-5.

## Introduction

Lumbar disc herniation (LDH) is a common spinal disorder that usually results in pain and dysfunction [1]. Among the various surgical approaches available, full endoscopic transforaminal discectomy (FETD) has gained popularity as a minimally invasive technique that offers potential advantages such as reduced tissue trauma and faster recovery [2, 3].

Obesity, characterized by excessive accumulation of adipose tissue, has been recognized as a significant factor affecting the natural history and treatment outcomes of various musculoskeletal conditions [4, 5]. Given the intricate anatomical considerations in the lumbar spine and the potential implications of increased adiposity on surgical access and healing processes, understanding the interaction between obesity and the results of FETD is crucial to optimizing patient care [6, 7].

While individual studies have investigated the association between obesity and FETD outcomes [8–10], a comprehensive evaluation of the current evidence is warranted. Systematic reviews and meta-analyses offer a robust approach to synthesize existing knowledge and identify key trends. This systematic review aims to critically appraise the relevant literature comparing clinical outcomes of FETD in obese and non-obese patients. Specifically, we seek to elucidate the impact of obesity on pain relief, functional improvement, and complication rates following FETD. By examining the collective evidence, we strive to identify potential outcome differences that can inform clinical decision-making and ultimately improve patient care.

## Methods and materials

### Study strategy

A systematic and comprehensive search was conducted across prominent scholarly databases, including PubMed, Embase, Scopus, Web of Science, China’s National Knowledge Internet (CNKI) and Wanfang Data, adhering meticulously to the Preferred Reporting Items for Systematic Reviews and Meta-Analyses (PRISMA) guidelines [11, 12]. The search was executed on November 18, 2023, employing a set of keywords including “lumbar disc herniation”, “endoscopic,” “transforaminal” and “obesity,” ensuring a comprehensive and focused exploration of the existing literature landscape.

To enhance the comprehensiveness of the search strategy, a secondary examination of the references cited in the selected articles was carried out, further fortifying the breadth and depth of the literature review.

### Inclusion and exclusion criteria

#### Inclusion criteria

(1) Original research articles with a quantitative research design, including randomized controlled trials (RCTs), cohort studies, and case-control studies. (2) Patients diagnosed with lumbar disc herniation who underwent FETD. Studies explicitly comparing clinical outcomes between obese and non-obese individuals following FETD. (3) Studies reporting on at least one of the following outcomes: pain relief, functional improvement, or complication rates. (4) Articles published in English or Chinese.

#### Exclusion criteria

(1) Reviews, editorials, letters, and conference abstracts. (2) Studies that included patients who had undergone a full endoscopic interlaminar discectomy or biportal endoscopic surgery. (3) Studies lacking a direct and clear comparison between obese and non-obese cohorts following FETD. (4) Studies without relevant and specific data on pain relief, functional improvement, or complication rates related to FETD.

### Data extraction

Two authors were assigned the responsibility of meticulously screening all retrieved articles resulting from the systematic search. In instances where conflicts occurred during the screening process, a judicious approach was taken by consulting the other coauthors. The resolution of discrepancies was achieved through collaborative discussion, ensuring a consensus reflecting the collective expertise of the research team [13]. The selection process involved a meticulous evaluation of the titles and abstracts to discern their relevance to the specific parameters of our study. In cases where ambiguity persisted or the information provided in the titles and abstracts proved insufficient, a comprehensive examination of the full-text articles was carried out. This rigorous approach aimed to determine the eligibility of studies based on predetermined inclusion and exclusion criteria.

The extracted data were meticulously classified into two discrete sections, each serving as a distinct focal point for subsequent analytical endeavors. The initial section of data extraction encompassed fundamental details related to the baseline characteristics of the included studies. This included key information such as the author’s name, year of publication, journal name, study design, gender distribution, sample size, and mean age of the patient cohort. The second component is the important clinical outcomes. This included the duration of surgery, hospitalization period, complication rates, Visual Analog Scale (VAS) scores, Oswestry Disability Index (ODI) scores, and MacNab results. In particular, complications were subcategorized into immediate and late postoperative occurrences. Furthermore, the evaluation of clinical indicators, represented by VAS and ODI scores, was conducted to define improvements at 3 months postoperatively and during the final follow-up, respectively.

### Quality assessment and publication bias

The methodological rigor of the studies incorporated into this meta-analysis underwent a comprehensive evaluation utilizing established tools, notably the Newcastle-Ottawa Scale (NOS) for non-randomized studies [14]. NOS facilitated a systematic evaluation of the quality of the study by assessing key parameters, including selection, comparability, and outcome, with studies achieving or exceeding a predetermined threshold of five “stars” deemed to meet high-quality criteria based on the specified rating criteria.

To further enhance the robustness of the synthesized evidence, this meta-analysis employed the Grading of Recommendations Assessment, Development, and Evaluation (GRADE) method [15]. The GRADE methodology systematically evaluated the credibility of the evidence derived from the pooled results. This evaluation considered several factors, including the risk of publication bias, the precision of the results, and the magnitude of the effects of treatment. The resulting quality of the evidence was then stratified into four hierarchical grades: high, moderate, low, and very low.

### Statistical analysis

Statistical meta-analyses were executed using the Review Manager 5.3 software, employing rigorous analytical methods to synthesize the available evidence. For continuous data, the weighted mean differences (WMD) computation accompanied by 95% confidence intervals (CI) was performed. Dichotomous outcomes were represented as odds ratios (OR) along with their corresponding 95% CL. The I^2^ statistic was employed to quantify the extent of heterogeneity, with a threshold of I^2^ ≥ 50% indicative of substantial heterogeneity. In instances where there was no discernible statistical heterogeneity (*P* > 0.1, I^2^ < 50%), a fixed-effects model was applied for the purpose of pooling. Conversely, in the presence of significant heterogeneity (*P* < 0.1, I^2^ ≥ 50%), a random-effects model was employed. The criterion for statistical significance was established at *P* < 0.05.

Furthermore, to examine the presence of publication bias, funnel plots were incorporated into the analysis. These graphs served as visual aids to detect asymmetry, providing information on the potential influence of publication bias on observed results.

## Results

### Search results and study characteristics

**Fig. 1:**
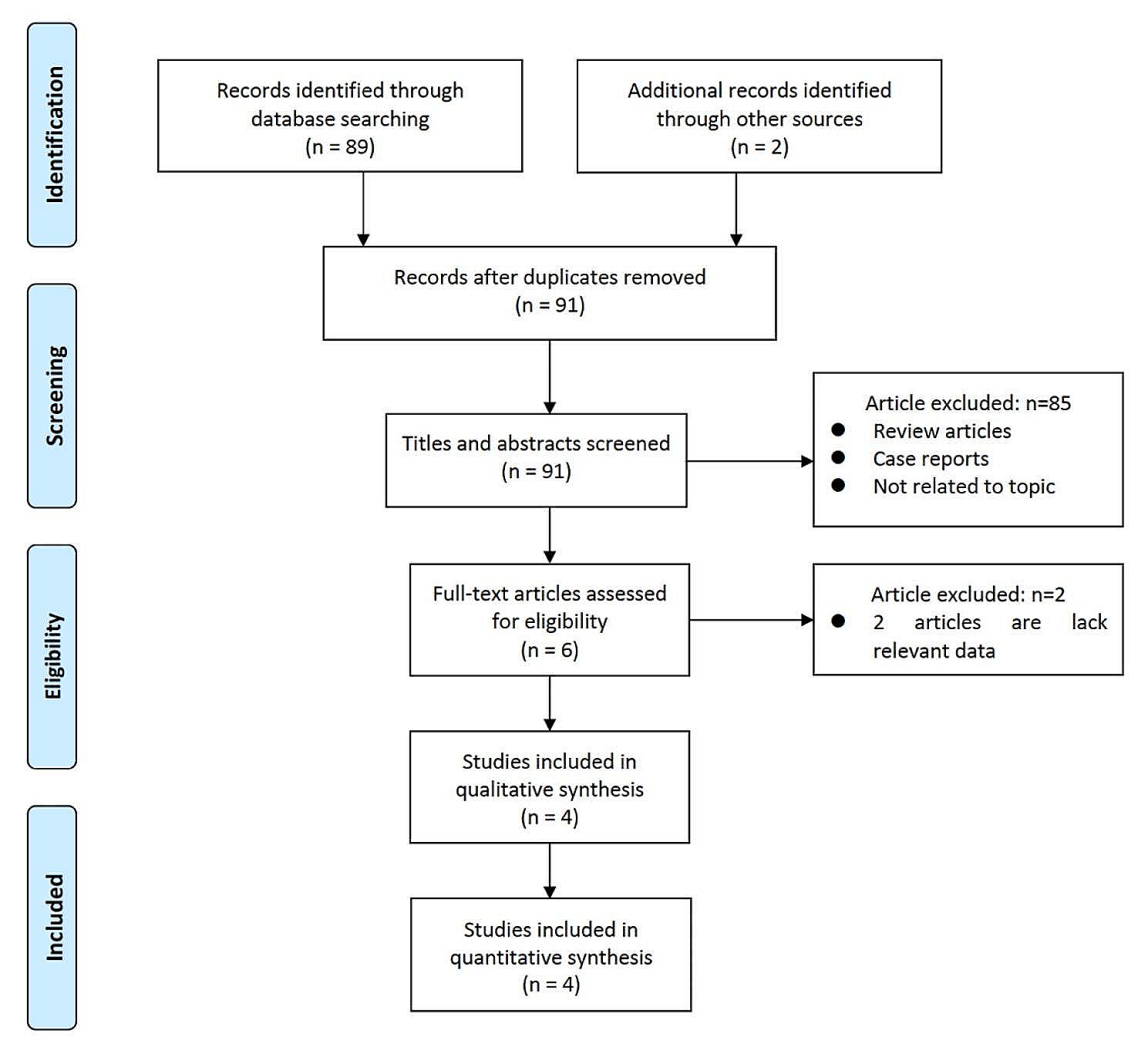
Flowchart of study selection for meta-analysis

Following a systematic and exhaustive literature search, a judicious selection process led to the identification of four studies that unequivocally met the predetermined inclusion criteria, as defined in Fig. 1. In particular, all four studies included in this analysis were retrospective in design [16–19].

The collective study cohort comprised a total of 258 patients, with 99 individuals assigned to the obese group and 159 to the non-obese group (Table 1). Among the selected studies, two originated in China, one from Korea, and the remaining one study from Greece. Within this amalgamated cohort, the treatment level that most frequently underwent FETD was identified as L4-5. In three studies, individuals with a body mass index (BMI) ≥ 30 were categorized as obese, whereas another study used more stringent criteria and categorized individuals with a BMI ≥ 40 as obese.

**Table 1:** Characteristics of the included studies

Study (Year)	Study Design	Country	Group (BMI)	Mean BMI (range), kg/m^2^	Sex (M/F)	Number	Mean Age (range), years	Operation level	Mean follow-up (range), months
Bae 2016^[16]^	Retrospective	Korea	Obese (≥ 30)	32.9 (30.1–38.9)	14/7	21	37.8 (20–60)	L2–3(2); L3–4(1); L4–5(13); L5–S1(5)	28.3 (24–43)
			Non-obese (18.5–22.9)	20.8 (19.2–22.8)	12/15	27	38.1 (17–75)	L4–5(21); L5–S1(6)	
Kapetanakis 2018^[17]^	Retrospective	Greece	Obese (≥ 40)	45.8 ± 4.5	8/12	20	57.3 ± 1.8	L3–4(1); L4–5(13); L5–S1(6)	24
			Non-obese (19.7–21.9)	20.8 ± 1.1	6/4	10	57.5 ± 1.8	L4–5(7); L5–S1(3)	
Yu 2021^[18]^	Retrospective	China	Obese (≥ 30)	NR	24/4	28	18.07 ± 2.21	L4–5(21); L5–S1(6);Other level (1)	27.75 ± 15.40 (12–70)
			Non-obese (< 30)	NR	66/14	80	18.16 ± 2.61	L4–5(50); L5–S1(28); Other level (2)	29.71 ± 16.45 (12–70)
Zhu 2021^[19]^	Retrospective	China	Obese (≥ 30)	NR	NR	30	NR	NR	12
			Non-obese (18.5–22.9)			42			

NR: not report; BMI: Body Mass Index.

### Perioperative measurements

#### Mean operative time (mins)

**Fig. 2:**
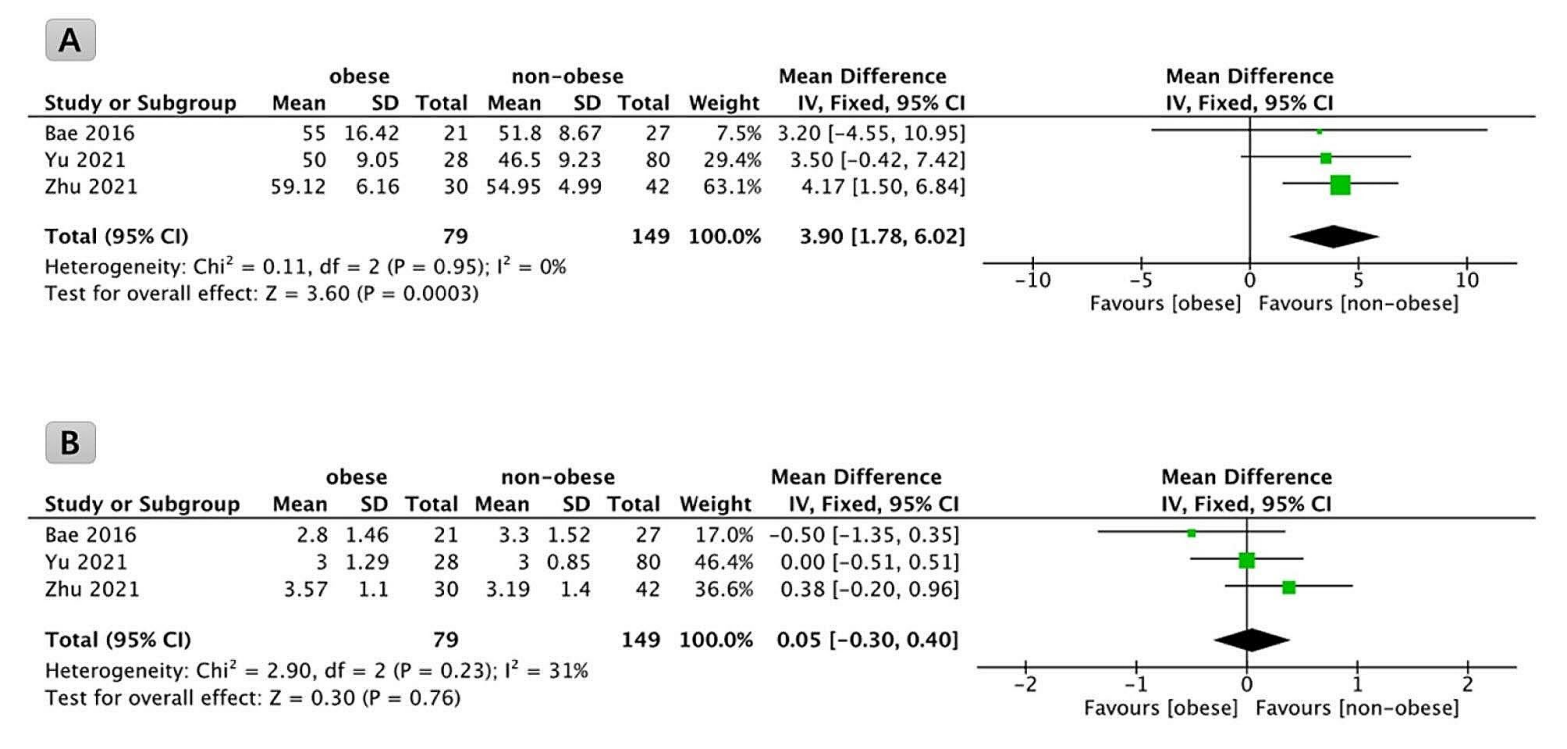
Forest plot comparison of operative time (**A**) and hospitalization (**B**) in obese and non-obese patients undergoing full endoscopic transforaminal discectomy

Incorporating data from three studies and a collective subject pool of 228 participants, an analysis of mean operative time was conducted. The findings revealed a statistically significant distinction in surgical duration between obese and non-obese patients. Specifically, obese patients exhibited a prolonged operative time compared to their non-obese counterparts (*P* = 0.0003, WMD: 3.90; 95% CI: 1.78 to 6.02, Fig. 2A).

#### Hospital length of stay (days)

A total of 228 subjects across the three studies provided the length of hospitalization. The analysis showed no difference in the length of hospitalization after FETD surgery between obese and non-obese patients (*P* = 0.76, WMD: 0.05; 95% CI: -0.30 to 0.40, Fig. 2B).

### Clinical outcomes

#### Improvement of VAS

**Fig. 3:**
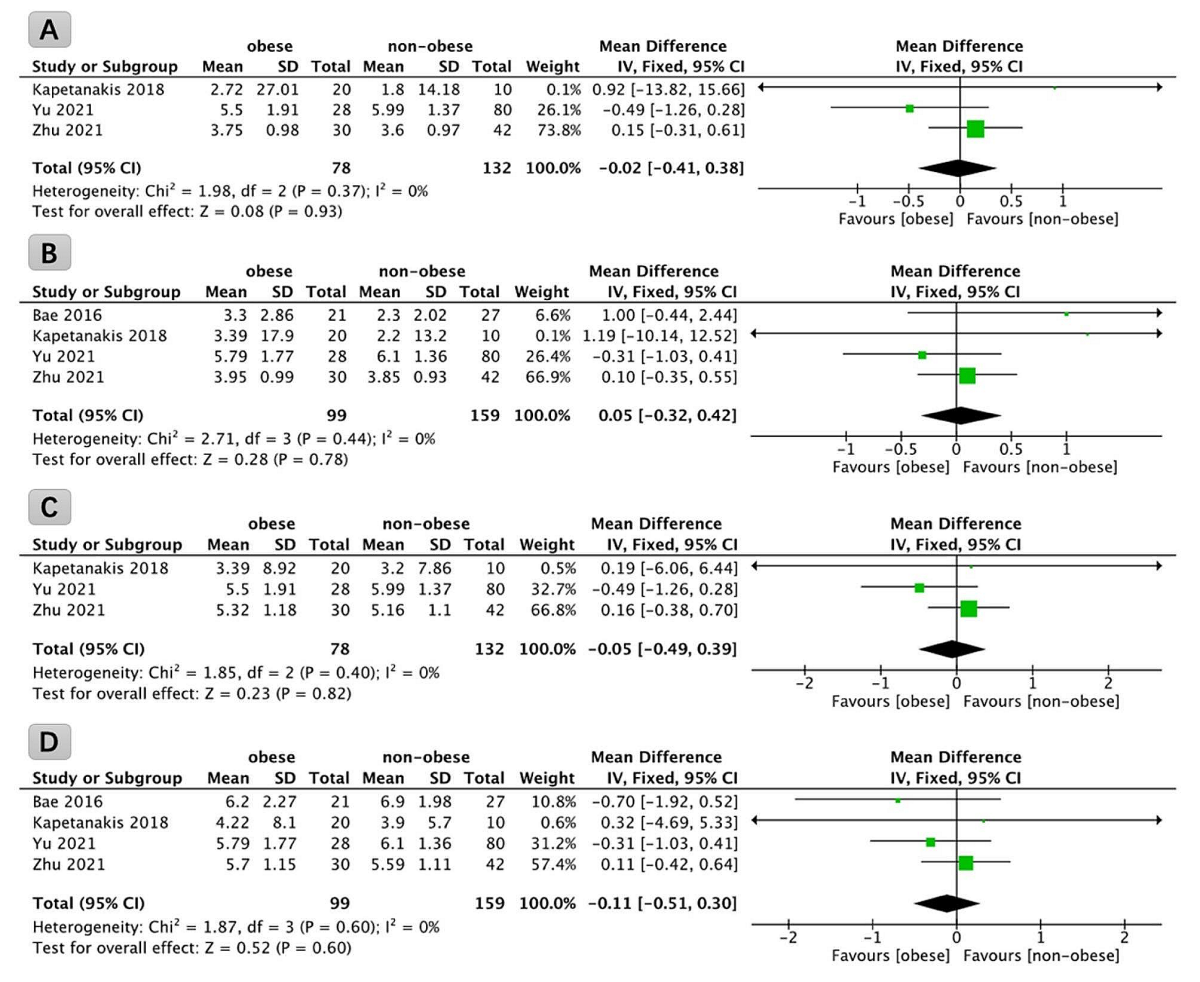
Forest plot comparing the improvement of VAS scores for back pain at 3 months (**A**), back pain at final follow-up (**B**), leg pain at 3 months (**C**), and leg pain at final follow-up (**D**) in obese and non-obese patients undergoing full endoscopic transforaminal discectomy. VAS: visual analog scale

Three studies reported VAS for back pain scores in 210 patients preoperatively and at 3 months postoperatively. The results of the analyses did not show statistically significant differences in the improvement of VAS for back pain at 3 months after FETD in obese patients compared to non-obese patients (*P* = 0.93, WMD: -0.02; 95% CI: -0.41 to 0.38, Fig. 3A).

A comprehensive analysis incorporating data from four studies, involving a total of 258 patients, was conducted to evaluate VAS for back pain before surgery and at the final follow-up. The results of this meta-analysis indicated that the improvement in back pain VAS scores at the final follow-up, after FETD, did not show statistically significant differences between obese and non-obese patients (*P* = 0.78, WMD: 0.05; 95% CI: -0.32 to 0.42, Fig. 3B).

Three studies reported VAS for leg pain scores in 210 patients preoperatively and at 3 months postoperatively. The results of the analyses showed no statistically significant difference in the improvement of VAS for leg pain at 3 months after FETD in obese patients compared with non-obese patients (*P* = 0.82, WMD: -0.05; 95% CI: -0.49 to 0.39, Fig. 3C).

A comprehensive analysis incorporating data from four studies, involving a total of 258 patients, was conducted to evaluate VAS for leg pain before surgery and at the final follow-up. The results of this meta-analysis indicated that the improvement in leg pain VAS scores at the final follow-up, subsequent to FETD, did not exhibit statistically significant differences between obese and non-obese patients (*P* = 0.60, WMD: -0.11; 95% CI: -0.51 to 0.30, Fig. 3D).

#### Changes in ODI

**Fig. 4:**
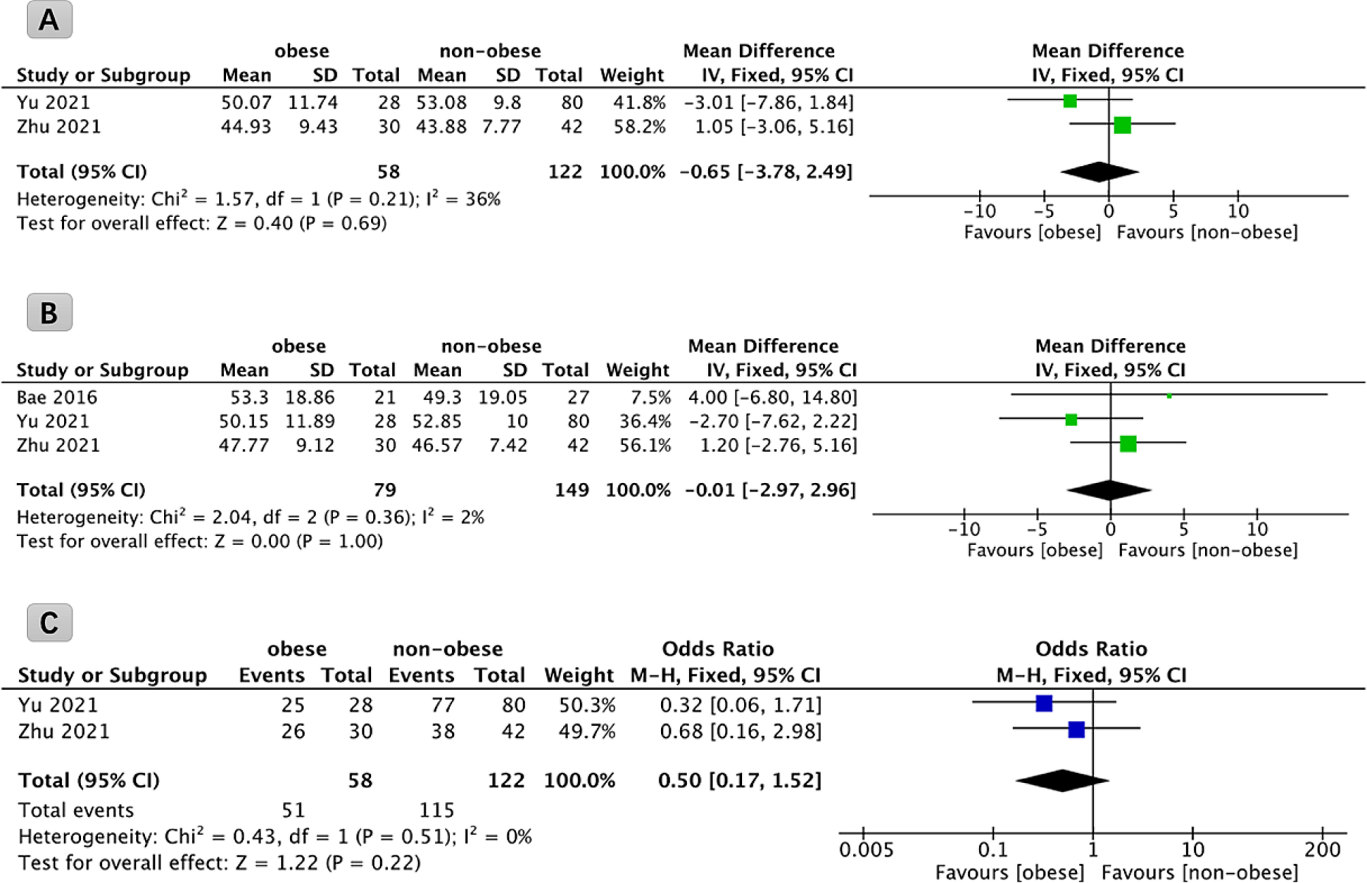
Forest plot comparing the improvement of ODI scores at 3 months (**A**), at final follow-up (**B**), and the final satisfaction rate (**C**) in obese and non-obese patients undergoing full endoscopic transforaminal discectomy. ODI: Oswestry Disability Index

Two studies reported ODI scores in 180 patients preoperatively and 3 months postoperatively. The results of the analyses did not show statistically significant differences in the improvement in ODI at 3 months after FETD in obese patients compared to non-obese patients (*P* = 0.69, WMD: -0.65; 95% CI: -3.78 to 2.49, Fig. 4A).

A comprehensive analysis was performed that included data from three studies, involving a total of 228 patients, to evaluate ODI preoperatively and at the final follow-up. The results of this meta-analysis indicated that the improvement of ODI at the final follow-up, subsequent to FETD, did not exhibit statistically significant differences between obese and non-obese patients (*P* = 1.00, WMD: -0.01; 95% CI: -2.97 to 2.96, Fig. 4B).

#### Satisfaction

Surgical satisfaction, assessed through the modified MacNab criteria, was evaluated in two studies encompassing a cohort of 180 patients. The meta-analysis of these results indicated that there was no statistically significant difference in the final satisfaction between obese and non-obese patients following FETD (*P* = 0.22, WMD: 0.50; 95% CI: 0.17 to 1.52, Fig. 4C).

### Complications

**Fig. 5:**
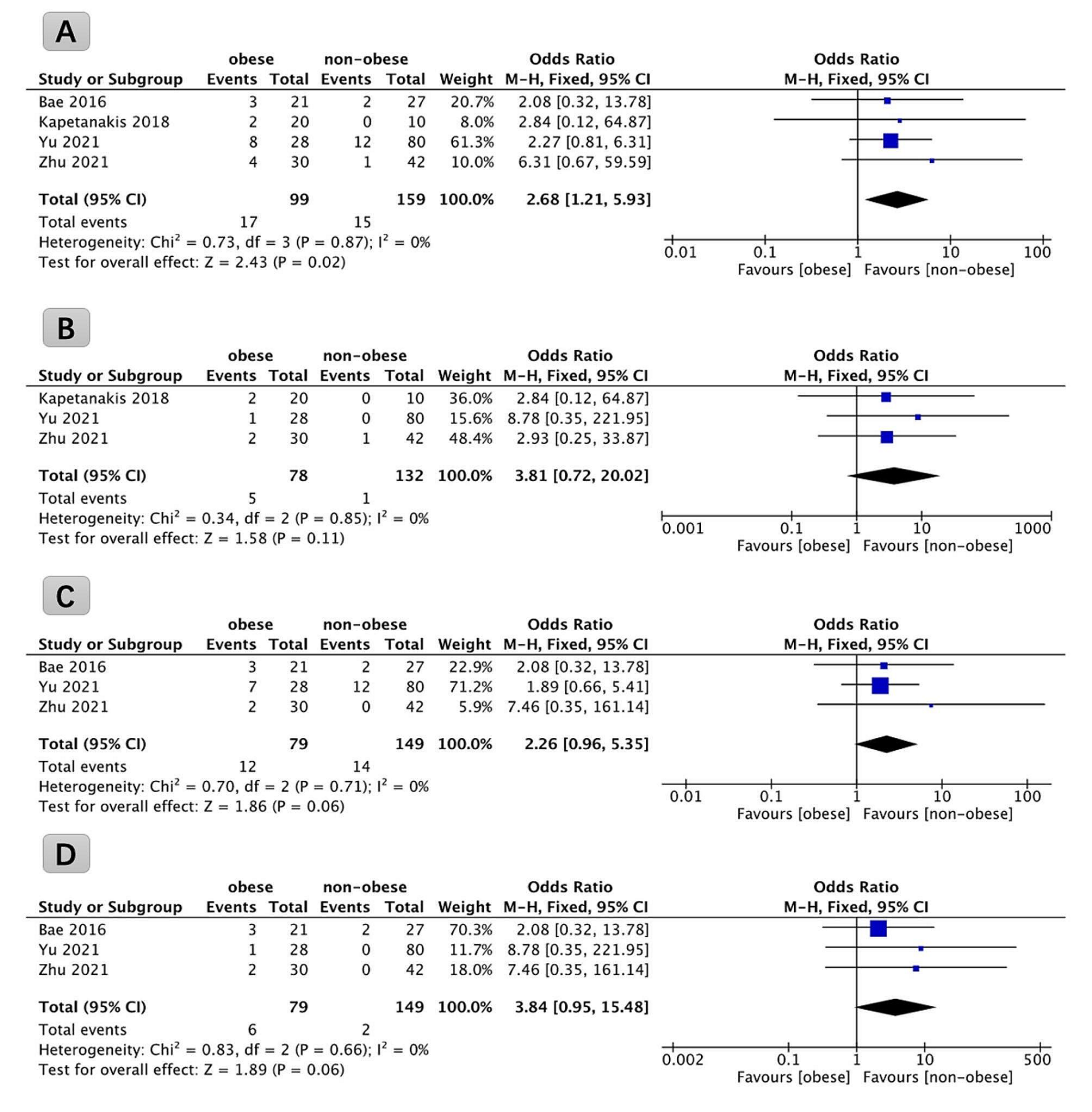
Forest plots comparing total complication rate (**A**), immediate complication rate (**B**), late complication rate (**C**), and recurrence rate (**D**) in obese and non-obese patients undergoing full endoscopic transforaminal discectomy

Data pertaining to postoperative complications from four studies, encompassing a collective cohort of 258 patients, were systematically analyzed. The results of this meta-analysis revealed that obese patients exhibited a higher incidence of total complications after FETD compared to their non-obese counterparts (*P* = 0.02, OR: 2.68; 95% CI: 1.21 to 5.93, Fig. 5A). The incidence of total complications within the obese group was documented at 17.17%, while the non-obese group exhibited a lower rate of 9.43%.

Data pertaining to immediate complications were derived from three studies, encompassing a cohort of 210 patients, and subjected to systematic analysis. The findings of this meta-analysis indicated that the rate of immediate complications lacked a statistical distinction between non-obese and obese patients undergoing FETD (*P* = 0.11, OR: 3.81; 95% CI: 0.72 to 20.02, Fig. 5B). However, the incidence of immediate complications within the obese group was documented at 6.41%, while the non-obese group exhibited a lower rate of 0.76%.

Data pertaining to late complications were derived from three studies, encompassing a cohort of 228 patients, and subjected to systematic analysis. The findings of this meta-analysis indicated that the rate of late complications lacked statistical distinction between non-obese and obese patients undergoing FETD (*P* = 0.06, OR: 2.26; 95% CI: 0.96 to 5.35, Fig. 5C). Nevertheless, the incidence of late complications within the obese group was documented at 15.19%, while the non-obese group exhibited a lower rate of 9.39%.

Furthermore, an examination of postoperative recurrences was conducted, utilizing data from three studies comprising a total of 228 patients. The meta-analysis revealed that the recurrence rate subsequent to FETD was comparable between non-obese and obese patients, with no statistically significant difference observed (*P* = 0.06, OR: 3.84; 95% CI: 0.95 to 15.48, Fig. 5D). Interestingly, the incidence of recurrences within the obese group was documented at 7.59%, while the non-obese group exhibited a lower rate of 1.34%.

### Others

The study conducted by Bae et al. [16] found that, the quantity of removed disc material during the FETD procedure was 0.9 cc, with a range of 0.5–2 cc, for the obese patients. In comparison, non-obese patients had a slightly higher amount of disc material removed, measuring 1.4 cc within a similar range of 0.5–2 cc.

The study conducted by Zhu et al. [19] reported that, within their investigated cohort, the obese group of patients exhibited higher values in terms of the number of intraoperative fluoroscopies, access establishment time, and procedure time in comparison to the non-obese group (all *p* < 0.05).

### Quality analysis and publication bias

Table 2 presents a comprehensive overview of the risk of bias assessment conducted for all studies included in the meta-analysis. In particular, each study exceeded a predetermined quality threshold, as evidenced by NOS scores of 5 stars or more. This consistent high-quality scoring across studies attests to the robustness of the evidence synthesized in this meta-analysis.

**Table 2:** Quality assessment of the included studies

Studies	Selection				Comparability	Exposure			Total scores (of 9)
	Is the case definition adequate?	Representativeness of the cases	Selection of Controls	Definition of Controls	Comparability of cases and controls on the basis of the design or analysis	Ascertainment of exposure	Same method of ascertainment for cases and controls	Non-Response rate	
Bae 2016^[16]^	☆	☆			☆☆	☆	☆	☆	7
Kapetanakis 2018^[17]^	☆	☆			☆☆	☆	☆		6
Yu 2021^[18]^	☆	☆			☆☆	☆	☆	☆	7
Zhu 2021^[19]^	☆	☆			☆	☆	☆		5

**Fig. 6:**
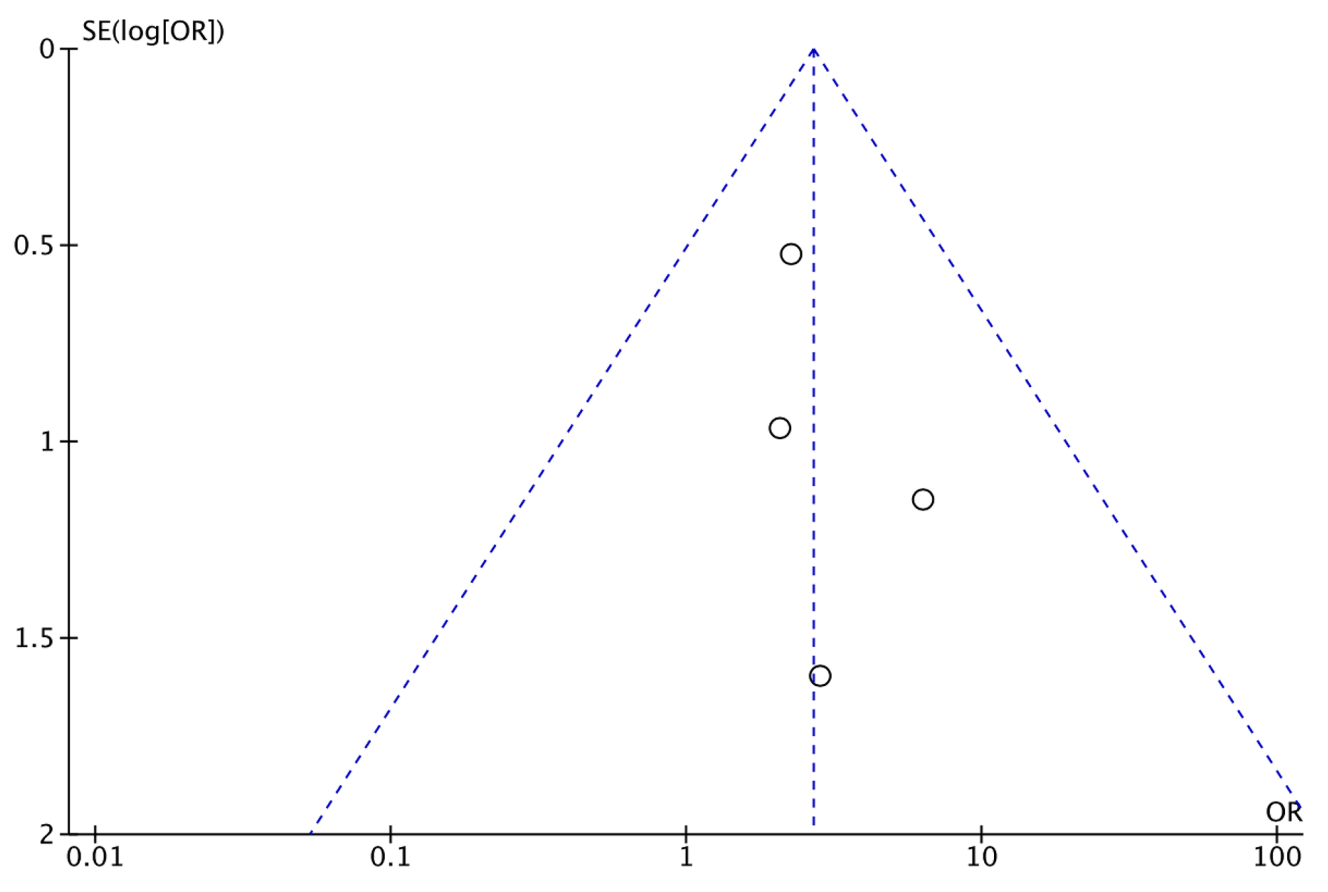
Funnel plot of publication bias for complications

To further scrutinize the potential for publication bias, particularly in the context of overall comorbidity, a visual inspection of the funnel plot (Fig. 6) was undertaken. The symmetrical distribution observed within the funnel plot suggests a low risk of publication bias, enhancing the credibility of the findings.

Table 3 provides a concise summary of the GRADE methodology employed to assess confidence in the overall results.

**Table 3:** A credibility assessment according to the GRADE scoring system

Outcome	No. of participants (studies)	Quality of the evidence (GRADE)	Comments and overall results
Mean operative time	228 (3)	High	The findings revealed a statistically significant distinction in surgical duration between obese and non-obese patients. Specifically, obese patients exhibited a prolonged operative time compared to their non-obese counterparts (*P* = 0.0003)
Improvement of VAS	258 (4)	Moderate	The results of the analyses did not show statistically significant differences in the improvement of VAS for back and leg pain in obese patients compared to non-obese patients after FETD (*P* > 0.05)
Changes in ODI	228 (3)	Moderate	The results of the analyses did not show statistically significant differences in the improvement of ODI in obese patients compared to non-obese patients after FETD (*P* > 0.05)
Satisfaction rate	180 (2)	Moderate	The meta-analysis of these results indicated that there was no statistically significant difference in the final satisfaction between obese and non-obese patients following FETD (*P* = 0.22)
Complication rate	258 (4)	Moderate	The results of this meta-analysis revealed that obese patients exhibited a higher incidence of total complications after FETD compared to their non-obese counterparts (*P* = 0.02)
Recurrence rate	228 (3)	Low	The meta-analysis revealed that the recurrence rate subsequent to FETD was comparable between non-obese and obese patients, with no statistically significant difference observed (*P* = 0.06)

VAS: visual analog scale; ODI: Oswestry Disability Index; FETD: full endoscopic transforaminal discectomy

## Discussion

We performed this analysis to determine the perioperative variables and postoperative clinical outcomes of obese patients receiving FETD. We observed that obese patients had significantly longer mean operative times and higher overall postoperative complication rates compared with non-obese patients. The identified differences in operative times and complication rates underscore the potential challenges and considerations inherent in FETD procedures in obese individuals. The extended operative times may be indicative of increased technical complexity, possibly attributed to anatomical variations or procedural intricacies associated with obesity. In addition, the epidural fat popping out early in the surgery needs more fluid for the field to remain clearer. So, this increases the time and also chances of prodrome. Moreover, the higher overall postoperative complication rates in obese patients emphasize the need for heightened vigilance and tailored perioperative management strategies to address potential challenges and enhance patient safety. It should be noted that this meta-analysis represents the inaugural comparative examination of clinical outcomes in the context of obese versus non-obese patients with LDH undergoing FETD. This analysis not only informs clinical decision-making but also serves as a foundational reference for future investigations aimed at refining and optimizing surgical strategies for LDH in the context of obesity.

Beyond its well-established association with cardiovascular and cerebrovascular diseases, obesity significantly contributes to orthopedic ailments [20–22]. While prior orthopedic literature primarily focused on weight-bearing knee degenerative diseases in obese patients [23], recent advancements in spinal biomechanics have unveiled a substantial linear correlation between obesity and conditions such as low back pain and lumbar disc herniation [24, 25]. International research has highlighted that severely obese individuals experience 1.5 times greater lumbar forces than normal, requiring increased lumbar back muscle exertion to maintain body balance and prevent deviation from the central axis [26]. This increased lumbar force increases the risk of lumbar strain or disc herniation. In addition, the substantial load borne by the lower lumbar intervertebral discs in severely obese patients exacerbates degeneration, making the nucleus pulposus and annulus fibrosus more susceptible to rupture under equivalent external forces [27]. Furthermore, the likelihood of vascular sclerosis or injury of the upper and lower endplate in severely obese individuals contributes to impediments in the supply of nutrients, resulting in metabolic imbalances, reduced matrix synthesis, increased acid metabolites, and diminished water content in the nucleus pulposus [8, 28]. Together, these factors contribute to the appearance of disc herniation and pose challenges for postoperative disc repair. Consequently, the judicious and effective use of surgery in the treatment of disc herniation in severely obese patients has become a focal point of discussion within the realm of spinal surgery.

Traditional open laminectomy serves as a prevalent clinical intervention for LDH, effectively mitigating mechanical compression within the spinal canal. However, the inherent drawbacks of the procedure, such as excessive manipulation of the paravertebral muscles that leads to a greater risk of hemorrhage and an increased incidence of postoperative adhesion, warrant consideration [8]. Moreover, the utilization of general anesthesia in traditional procedures introduces elevated anesthesia-associated risks, prolonged postoperative recovery, and heightened susceptibility to complications like urinary tract infections and pneumonia [29]. In contrast, FETD offers a distinct approach. The employment of local anesthesia during FETD not only diminishes anesthesia-related risks but also facilitates direct communication with the patient, reducing the likelihood of neurological damage. FETD obviates the need for spinal cord retraction and bone removal, minimizing the impact on adjacent soft tissues and muscles [16]. Precise decompression through a small incision maximizes the preservation of posterior spinal integrity and mitigates potential complications [17]. In addition, this approach has minimal impact on the feasibility of subsequent posterior decompression or fusion surgery.

FETD offers several theoretical advantages for obese patients undergoing surgical intervention for LDH. FETD utilizes a small surgical incision, potentially reducing the incidence of incisional fatty liquefaction, a complication more common in obese patients due to the increased adipose tissue at the incision site. Discography can be performed concurrently with FETD, allowing for precise localization of the ruptured annulus fibrosus, the source of pain in LDH. This combined approach can enhance diagnostic accuracy compared to traditional methods. Staining the surgical field with a methylene blue and iodine alcohol mixture can improve visualization of anatomical structures, particularly nerve roots. This improved visualization can facilitate a smoother and more efficient surgical procedure. The use of a radiofrequency knife during FETD offers potential benefits. It may ablate nerve endings that have infiltrated the ruptured annulus fibrosus, potentially reducing post-operative pain. Additionally, the radiofrequency technology may lessen the formation of nerve adhesions, a potential source of chronic pain. Continuous saline irrigation throughout the procedure can effectively flush out chemical irritants released from the ruptured disc material. This reduces the accumulation of these substances and minimizes potential chemical irritation of the nerve root, potentially leading to faster recovery and reduced post-operative pain. Despite its effectiveness, FETD comes with a steeper learning curve, narrower indications compared to traditional open surgery, limited decompression capability, longer working channels required for obese patients, and the imperative need for weight control and avoidance of early postoperative physical exertion.

Comprehensive meta-analysis of three and four studies evaluating VAS for back pain and leg pain scores, and ODI before and after surgery, at 3 months postoperatively, and at the final follow-up, revealed no statistically significant differences in improvement between obese and non-obese patients after FETD. The findings indicate that surgical outcomes in terms of pain relief and functional improvement were comparable between the two groups at both short-term and long-term follow-up. Furthermore, the evaluation of surgical satisfaction using the modified MacNab criteria did not show significant differences between obese and non-obese patients. Collectively, these results suggest that FETD yields similar clinical benefits in terms of pain relief, functional improvement, and patient satisfaction, regardless of the obesity status.

Our analysis revealed a significantly higher overall complication rate in obese patients compared to their non-obese counterparts. Interestingly, however, the rates of immediate and late complications did not differ statistically between the two groups. Common surgical complications associated with FETD include various challenges, including postoperative radicular sensory abnormalities, dural tears, and nerve root injuries. Among these, postoperative radicular sensory abnormalities emerge as the most common complication, often attributed to overstimulation or nerve root damage. Prudent preoperative evaluation and a gentle operative approach are crucial in mitigating the risk of this complication [30]. Dural tears, often encountered in inexperienced practitioners or cases with substantial subdural scar tissue adhesion, underscore the importance of meticulous and cautious surgical procedures to prevent iatrogenic injury. Nerve root injuries, the most serious complication, often result from insufficient familiarity with anatomical structures and inadvertent maneuvers, emphasizing the need for careful identification of tissue structures and avoidance of rough operative techniques. Recurrence rates after FETD, reported in the literature ranging from 5 to 15% [31], highlight the significance of thorough removal of degenerated intervertebral disc tissue during the operation to minimize the risk of recurrence. Postoperatively, lumbar back muscle exercises and a month-long protective regimen against heavy physical labor are recommended to further reduce the likelihood of recurrence. While FETD proves to be an effective, safe, and minimally invasive surgical method for lumbar disc herniation treatment, careful adherence to surgical indications and contraindications, along with continual improvement in surgical proficiency, remains crucial in minimizing the incidence of complications associated with this technique.

In addition, the findings of our study carry significant clinical implications, particularly for the management of obese patients undergoing FETD. Obesity poses unique challenges in surgical interventions due to increased surgical complexity, higher complication rates, and potentially inferior outcomes. Therefore, tailoring surgical approaches to address these challenges is crucial. Firstly, prolonged operative time can potentially increase the risk of intraoperative complications, such as anesthesia-related issues, surgical site infections, and blood loss. Clinicians should consider these factors when planning surgical schedules and allocating resources for obese patients undergoing FETD. Strategies to optimize perioperative management, such as meticulous surgical planning, preoperative optimization of comorbidities, and intraoperative monitoring, are essential to mitigate the increased risks associated with prolonged surgery. Secondly, given the higher prevalence of comorbidities and anatomical variations in obese patients, thorough preoperative assessment is crucial to identify potential risk factors and optimize surgical outcomes. This includes assessing the severity and duration of symptoms, evaluating the extent of disc herniation, and considering the presence of concomitant spinal pathologies. Clinicians should also take into account the patient’s body habitus, spinal anatomy, and overall health status when determining the suitability for FETD. Furthermore, we emphasize the need for tailored surgical techniques and instrumentation to address the anatomical challenges posed by obesity. This may involve using longer instruments, specialized retractors, and advanced imaging modalities to navigate through adipose tissue and reach the target disc space safely and effectively. Additionally, intraoperative fluoroscopy or navigation systems can aid in accurately localizing the surgical site and minimizing the risk of iatrogenic injury. Lastly, comprehensive postoperative care and rehabilitation in optimizing outcomes for obese patients undergoing FETD. Close postoperative monitoring for potential complications, such as wound infections, neurological deficits, and recurrence of symptoms, is essential in obese individuals due to their heightened susceptibility. Moreover, implementing tailored rehabilitation programs focusing on weight management, core stabilization, and lifestyle modifications can promote long-term success and prevent recurrence of disc herniation in obese patients.

### Limitations

Some limitations of the present study lie in the predominantly retrospective nature of the included studies (including selection bias, information bias, confounding variables, and challenges in establishing external validity), coupled with the relatively modest number of available studies, both of which contribute to the overall constraints of this review. Furthermore, the discernible divergence in the definitions of obesity between the respective authors of the included studies represents a notable weakness, introducing variability and potential bias into our findings. Despite these limitations, we contend that our study has yielded valuable information, providing a foundation for future investigations.

## Conclusion

The key findings of our meta-analysis underscore notable distinctions between obese and non-obese patients who undergo FETD. In particular, obese individuals exhibited prolonged durations in receiving FETD surgery and experienced a higher overall postoperative complication rate compared to their non-obese counterparts. However, no statistically significant differences were discerned between the two groups regarding the length of hospitalization, the extent of improvement in VAS scores, the improvement in ODI, and the recurrence rates. These findings, though noteworthy, merit consideration in the context of acknowledged limitations in study design and heterogeneity among included studies. Despite these constraints, our investigation serves as a valuable preliminary exploration and refine our understanding of surgical management strategies for LDH in both obese and non-obese patients.

### Electronic supplementary material

Below is the link to the electronic supplementary material.

Supplementary Material 1

## Data Availability

The datasets are presented within the manuscript.
